# Change and Stability in Political Ideology and Engagement over the Lifespan: A Retrospective Study

**DOI:** 10.1093/geroni/igaf122.3391

**Published:** 2025-12-31

**Authors:** Kyla Prussman, Elizabeth Rickenbach, Jennifer Lucas, Kevin Doran

**Affiliations:** Saint Anselm College, Goffstown, New Hampshire, United States; Saint Anselm College, Goffstown, New Hampshire, United States; Saint Anselm College, Goffstown, New Hampshire, United States; Saint Anselm College, Goffstown, New Hampshire, United States

## Abstract

Voters aged 60+ years old are historically important for US elections, due to their persistently high voting rates, the significance of Medicare and Social Security, and population aging (Binstock, 2012; Campbell & Binstock, 2011; Super, 2020). Research has long sought to understand older voter (OV) lifetime experiences with politics and the development of their political ideology across their lifetime. Most research on OV’s political ideology is cross-sectional and focuses on general polling data, and research on lifetime political experiences is often framed through a political science or economic lens. The current study incorporated a lifespan and psychological perspective with semi-structured interviews to retrospectively examine perspectives of OVs. In particular, the current study sought to understand whether political preferences vary over a person’s life and whether stability or change differs across gender or political party. Participants (n = 88) included New Hampshire registered voters aged 60+ years. The sample included 29 Independent voters (14 women, 15 men), 31 Democratic voters (16 women, 15 men), and 28 Republican voters (15 women, 13 men). Qualitative findings showed change and stability across OVs. Many voters’ shared stability across their lifespan and increased intensity towards their political engagement. Others discussed shifts from more conservative values to more liberal values, or from a lack of political engagement to more engagement. OVs discussed how their shifts were influenced by lifetime experiences with work, family, and health, as well as shifting perspectives towards politics. This research contributes to a better understanding of the dynamic nature of OV’s political views.

